# Upcoming imaging concepts and their impact on treatment planning and treatment response in radiation oncology

**DOI:** 10.1186/s13014-018-1091-1

**Published:** 2018-08-13

**Authors:** Paul Russell Roberts, Ashesh B. Jani, Satyaseelan Packianathan, Ashley Albert, Rahul Bhandari, Srinivasan Vijayakumar

**Affiliations:** 1 Department of Radiation Oncology, University of Mississippi Medical Center, 350 Woodrow Wilson Drive Suite 1600, Jackson, MS 39213 USA; 2 Department of Radiation Oncology, Winship Cancer Institute of Emory University, 1365 Clifton Rd, Atlanta, GA 30322 USA

**Keywords:** Radiation therapy, Treatment planning, Treatment response, Imaging

## Abstract

For 2018, the American Cancer Society estimated that there would be approximately 1.7 million new diagnoses of cancer and about 609,640 cancer-related deaths in the United States. By 2030 these numbers are anticipated to exceed a staggering 21 million annual diagnoses and 13 million cancer-related deaths. The three primary therapeutic modalities for cancer treatments are surgery, chemotherapy, and radiation therapy. Individually or in combination, these treatment modalities have provided and continue to provide curative and palliative care to the myriad victims of cancer.

Today, CT-based treatment planning is the primary means through which conventional photon radiation therapy is planned. Although CT remains the primary treatment planning modality, the field of radiation oncology is moving beyond the sole use of CT scans to define treatment targets and organs at risk. Complementary tissue scans, such as magnetic resonance imaging (MRI) and positron electron emission (PET) scans, have all improved a physician’s ability to more specifically identify target tissues, and in some cases, international guidelines have even been issued. Moreover, efforts to combine PET and MR to define solid tumors for radiotherapy planning and treatment evaluation are also gaining traction.

Keeping these advances in mind, we present brief overviews of other up-and-coming key imaging concepts that appear promising for initial treatment target definition or treatment response from radiation therapy.

## Background

Despite many advances, cancer continues to represent a significant health burden in the United States and the rest of the world. The American Cancer Society estimates that in 2018, approximately 1.7 million new diagnoses of cancer and about 609,640 cancer-related deaths are anticipated in the United States. Worldwide, cancer accounts for about 1 in 7 of all deaths. Given the far-reaching impact of cancer, affordable and remotely utilizable imaging technologies are needed to improve diagnostic accuracy, treatment efficacy, and the value of post-treatment surveillance in cancer care [1].

The three primary therapeutic modalities for cancer treatments are surgery, chemotherapy, and radiation therapy. Individually or in combination, these treatment modalities have provided and continue to provide curative or palliative care to the myriad victims of cancer. Of the three, it is radiation therapy which is most heavily dependent upon imaging technologies, often on a daily basis, if image-guided radiotherapy is used. Today, the primary modality through which any type of conventional photon radiation therapy is planned is based upon a computed tomography (CT) scan of the patient in the treatment position – broadly referred to as CT-based treatment planning. The prescribing, recording, and reporting of photon radiation treatments is governed by the recommendations in ICRU 50 and ICRU 62, two reports generated by the International Commission on Radiation Units and Measurements and used virtually worldwide (ICRU, 1978, 1991) [2, 3]. In radiation treatment planning, it is generally accepted that there are two major categories of tissues – the tissues which are to be specifically treated and the tissues to be avoided (also called the organs at risk or OARs). Hence, the precision with which we are able to avoid or minimize the dose to the OARs and maximize dose to the targeted tissues depends upon the accuracy with which the tissues which are to be treated and the OARs are defined upon each slice of the CT scan by the treating radiation oncologist and the skill of the dosimetrist in subsequently generating a treatment plan that is highly “conformal” – meaning that the treatment volume is well enclosed by the prescribed dose and the surrounding tissues are maximally spared [4].

Although CT remains the primary treatment planning modality, we have over the past several years, moved beyond solely using the CT scan to define our treatment targets and OARs – complementary tissue scans such as magnetic resonance imaging (MRI) and positron electron emission (PET) scans have all improved our ability to more specifically identify target tissues and international guidelines have even been issued in some instances [5–10]. Indeed, in the use of PET scans, it has been noted that radionucleotides other than the standard ^18^F-fluorodeoxyglucose, appear to have specificity for distinct tissues – for instance ^18^F-ethyl-tyrosine for high grade glioma or ^18^F-fluorocholine for prostate cancer [11, 12]. While efforts to combine PET and MR to define solid tumors for radiotherapy planning and treatment evaluation are ongoing, discussion of these efforts are beyond the scope of this paper and have been discussed thoroughly elsewhere [10]. Keeping these on-going advances in mind, we present below brief overviews of some of the other up-and-coming key imaging concepts that appear promising for initial treatment target definition for radiation therapy or to help assess treatment response after some type oncologic treatment, which would then help in identifying tissues which may need a different therapeutic approach to its eradication.

## Upcoming imaging concepts

### Autofluorescence

Autofluorescence is an imaging modality that exploits inherent fluorescence of native tissues to generate images. First, a tissue surface is illuminated by light of a single wavelength that is either reflected, back-scattered, or induces autofluorescence. Emitted autofluoresced light is detected by a camera and displayed onto a monitor for interpretation within the visible light spectrum [13]. Tissue fluorescence is determined by its molecular composition, so benign and malignant tissues can be differentiated based on their color and quantity of autofluorescence [14].

Systems implementing autofluorescence have already been incorporated into devices spanning several clinical applications, including bronchoscopy, colonic polyp evaluation, quantification of colonic inflammation, and intraoperative tumor evaluation [14, 15]. Bronchoscopes with autofluorescent capabilities are currently being evaluated in cancer and precancerous lesion detection. The sensitivity of autofluorescence seems to be greater than that of white light bronchoscopy, but results regarding specificity have been variable [13]. When discriminating between adenomas from hyperplastic colonic polyps, autofluorescence has shown high sensitivity and specificity at 89 and 81%, respectively. It has been suggested that larger-scale studies need to be conducted to determine the efficacy of autofluorescence in diagnosing colon neoplasms and other inflammatory diseases [15].

Advantages of autofluorescence technology include real-time image acquisition, intra-surgical tumor assessment, lack of need for exogenous fluorophores, wide field-of-view, and ability to quantify fluorescence for objective diagnostic information. Limitations of this technology include its shallow depth-of-field, low specificity, high false positive rate, high cost, increased exam time, interference from bodily fluids within surgical field, and changes in tissue autofluorescence secondary to treatment [13–15].

Potential radiotherapy applications of autofluorescence are likely initially to be in the imaging and monitoring of superficial tumors and superficial response to therapy, such as differentiation of choroidal pigmented lesions, evaluating skin response, particularly using optically-tracked hand-held platforms, and image analysis of pre-neoplastic lesions along the upper aerodigestive tract [16–22]. Autofluorescence may also have application to surgical margin analysis, such as for breast cancer [20].

### Near-infrared fluorescent imaging

**Fig. 1:**
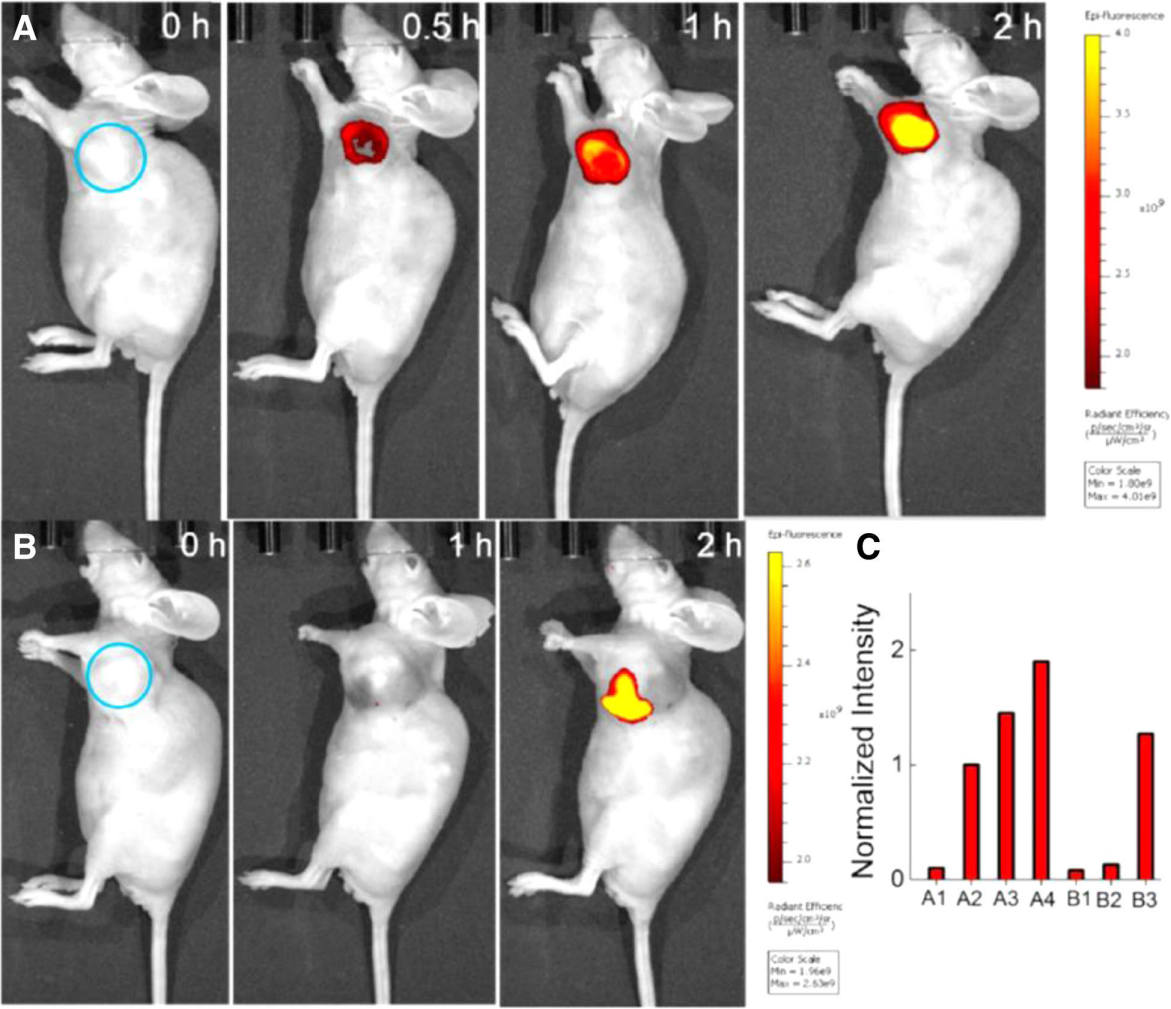
Fluorescent images of mice (pseudocolor). **a** Given an intratumoral injection of NALP (50 μL, 100 μM in a mixture of DPBS buffer (pH 7.4, 10 mM) and DMSO (4/1, *v*/v). **b** Before injection of NALP (50 μL, 100 μM in a mixture of DPBS buffer (pH 7.4, 10 mM) and DMSO (4/1, v/v), the tumor was pretreated with Na3VO4 (50 μL, 5 mM in doubly distilled water). **c** Quantification of imaging data, intensity in A-0.5 h was defined as 1. The selected circle region showed the tumor. (Used with permission. Originally published by Liu, et al. 2017 in In vivo imaging of alkaline phosphatase in tumor-bearing mouse model by a promising near-infrared fluorescent probe)

Near-infrared fluorophore (NIRF) imaging utilizes electromagnetic radiation within the near-infrared spectrum (700–900 nm) to generate images, rather than the visible light spectrum (390–700 nm) used by the naked eye [23]. Although large design variations exist between manufacturers, NIRF imaging devices operate on the same basic principles. First, a near-infrared fluorophore is injected into a subject. Second, fluorophore molecules accumulate in, near, or around the desired target and fluoresce. Third, photons within the near-infrared spectrum are detected by a receiver and displayed as a 2D image, which can be combined to form 3D images [22]. In Fig. 1, a near-infrared fluorescent probe accumulates within a murine tumor over time with subsequent quantification of fluorescent intensity.

The variety of dyes available determines the breadth of application of NIRF imaging. Both indocyanine green (ICG) and methylene blue (MB) are currently approved for clinical use by the FDA, while other dyes, such as CW800 and ZW800–1, have been used safely in animal studies but are not yet FDA approved for use in humans [23]. In the future, conjugating NIRFs to other molecules, such as antibodies, will allow a wider variety of targets to be imaged with greater resolution.

Per a recent review of upcoming imaging modalities, NIRF imaging may have the greatest potential for implementation in clinical practice in the near future [24]. Strengths of NIRF imaging include current FDA-approved imagers and dyes, deep depth-of-field (up to 4 cm), reduced image noise by native tissues and proteins, breadth of application with targeted dyes, and potential for real-time visualization [14, 23]. Current drawbacks of NIRF imaging are the limited number of FDA-approved dyes, time requirement to properly administer dyes, need of an imaging device to visualize fluorophores, and inability to overlay images with a surgical field, which functionally blinds surgeons during image generation [14, 23].

Potential radiotherapy applications of near-infrared fluorescent imaging are surgical guidance for solid tumors, including prostate cancer and breast cancer [24, 25]. Near-infrared fluorescent is a rapidly developing imaging technology that is quickly approaching clinical utility [26, 27]. It is a technology that is also likely applicable to real-time monitoring and modulation of external beam radiotherapy dose [28].

### Nonspecific fluorescent contrast agents

Nonspecific fluorescent markers exploit the Enhanced Permeation and Retention (EPR) effect to preferentially mark tumors. The EPR effect has two main components: enhanced permeation and retention. As tumors grow, they must form new blood vessels via angiogenesis to survive. These newly formed vessels have discontinuous basement membranes and fenestrations, which increase permeability of vessel contents compared to normal vasculature. This increased extravasation of vessel contents secondary to increased capillary permeability is known as the enhanced permeation aspect of the EPR effect. Normally macromolecules that have exited a vessel into the extracellular space are drained via the lymphatic system. In tumors, lymphatic drainage is dysfunctional, which results in an accumulation of extracellular macromolecules. This phenomenon is known as the retention aspect of the EPR effect. Taken together, tumor blood vessel contents, like nonspecific fluorescent contrast agents, are more easily spilled into the extracellular space (enhanced permeability) but remain there due to dysfunctional lymphatic drainage (retention) [29].

There are two nonspecific fluorescent contrast agents that are currently FDA-approved: indocyanine green (ICG) and methylene blue (MB). As previously discussed, the dyes CW800 and ZW800–1 have been used safely in animal studies but are not currently FDA-approved for use in humans.

Advantages of nonspecific fluorescent contrast agents are their rapid hepatic clearance and low toxicity. Disadvantages of these dyes are their relative contraindication in patients with contrast allergies, low signal-to-noise ratio, time-sensitive nature due to their rapid hepatic clearance, lack of intracellular accumulation, and extravasation during surgery [13].

Nonspecific fluorescent contrast agents that have been in development for many years and are now approaching clinical applications [30]. From the standpoint of radiotherapy applications, nonspecific fluorescent contrast agents are likely to have ubiquitous and versatile role, ranging from imaging for staging, imaging for guidance of radiotherapy planning, and imaging for radiotherapy treatment delivery.

### Targeted contrast agents

Cells within malignant tissues have abandoned much of the original tissue’s normal anatomy and physiology [29]. Genetic dysregulation leads to disorganized angiogenesis, aberrant cellular metabolism, and increased growth factor receptor expression [13]. The goal of targeted fluorescent probes is to exploit this distinct expression of various cellular components to better visualize malignant tissues. Targeted fluorescent agents accomplish this by pairing a highly specific ligand with a fluorescent reporting molecule. Ligands can be derived from antibodies, antibody fragments, proteins like transferrin, peptides, nucleic acids, and other small molecules [31]. Fluorescent molecules can either be constitutively active, or “activatable” [13, 29]. Previously, constitutively active fluorescent agents were discussed under the nonspecific fluorescent contrast agents section. “Activatable” probes are composed of quenched fluorophores coupled to enzyme substrates, which activate and fluoresce upon enzymatic cleavage. Since particular enzymes, such as metalloproteases, are upregulated in malignant cells, cancerous tissues preferentially fluoresce [30].

From the basic scientists’ bench to clinical trials, targeted contrast agents are at all stages of development. Currently, a few antibody fragment-based agents have made it to the clinical stages of testing, but the vast majority of agents await clinical validation [31].

Particular advantages of targeted contrast agents are their ability to use existing immunoglobulin-based therapies as ligands; significant depth-of-penetration by using NIRF imaging techniques; higher signal-to-noise ratio than nonspecific contrast agents; and wide variety of potential targets. Disadvantages of this technology are the requirement to use exogenous fluorescent agents; the intense amount of regulatory testing/validation required to reach clinical application; and low number of agents currently available for use [13, 29, 30].

Similar to nonspecific fluorescent contrast agents, targeted contrast agents are likely to have ubiquitous applications in cancer diagnosis and staging, as well as radiotherapy planning and delivery [32, 33]. As these contrast agents are typically targeted towards specific ligands, applications will likely be highest yield in tumors where the immunogenic targets have most clearly been identified.

### Radiofrequency spectroscopy

Radiofrequency spectroscopy (RS) generates images by measuring a sample’s unique interaction with electromagnetic radiation. Specifically, RS generates images by measuring the ability of a sample to move electrical charge (conductivity) and to store electrical charge (permittivity) within the radiofrequency range of the electromagnetic spectrum [13, 30, 34]. Cellular organization, size, morphology; intracellular and extracellular components; and membrane composition all influence a tissue’s conductivity and permittivity [34]. Since all of these properties can differ between benign and malignant tissues, their conductivity and permittivity can be used to determine the identity of a given sample [30, 34].

RS devices have been developed to evaluate both samples of breast and prostate tissues and surgical margins. In one intraoperative study of resected breast cancer, the use of an RS-based device was safe, effective, and showed the potential to decrease the rate of repeat operations [30, 35]. In ex vivo samples of resected prostate tissue, RS-based devices showed “strong potential for future use…[in]. differentiating benign and malignant prostate tissue” [34]

Strengths of radiofrequency spectroscopy include its ability to acquire images without external fluorophores; real-time imaging capability; and current existence of handheld devices. Weaknesses of this technology are its limited field-of-view and shallow depth-of-field. [13].

Radiofrequency spectroscopy is likely to have significant applications in radiotherapy, particularly in the detection of residual disease for the tailoring of intra-operative and post-operative radiotherapy [31]. Radiofrequency spectroscopy is also likely to assist in improving uniformity and homogeneity of diagnostic imaging for solid tumors, including prostate cancer [35].

### Raman spectroscopy

Raman spectroscopy is an “analytical optical spectroscopic” technique based on the Raman effect, a phenomenon first experimentally demonstrated in 1928, whereby the molecular components of a substance can be determined from their characteristic light scattering properties [36, 37]. In order to record a Raman spectrum, light of a single wavelength must first be directed at a given sample. Next, molecules within the sample enter an excited state as they absorb photons. Third, photons are emitted as molecules fall from higher to lower energy states. Most photons emitted are of the same energy that was originally absorbed; however, a fraction of photons is emitted at a different energy. The Raman spectrum is a measure of these energy shifts between photons [13]. The molecular composition of a sample can be determined when Raman spectra are measured across multiple wavelengths [38].

Raman spectroscopy’s utility is being appraised in a variety of clinical applications, including tissue biopsy evaluation, surgical margin assessment, metastatic lesion analysis, disease screening, and radiation treatment response. Devices are currently available in Canada, Europe, and Australia that can non-invasively diagnose skin cancer, while others are in development that can diagnose lung, colon, and cervical cancer [37]. In the lab, Raman spectroscopy has been used to differentiate between benign and malignant nervous tissue [39]. Elsewhere, this technology has been used to assess the response of malignant tissue to radiation [40]. Lastly, Raman spectroscopy has displayed the capability of transcutaneous blood glucose sensing [41].

Raman spectroscopy has immense potential to impact many aspects of medicine. Strengths of this technology fall within three major areas: ability to objectively identify tissue samples; real-time assessment of tumor progression and margins; and ability to determine tumor response to radiation treatment [30, 37]. However, this technology also has its shortcomings. First, conventional Raman spectroscopy is unable to assess deep seated tissues [37]. Other problems include signal interference from native tissues and thermal damage to measured material [38].

**Fig. 2:**
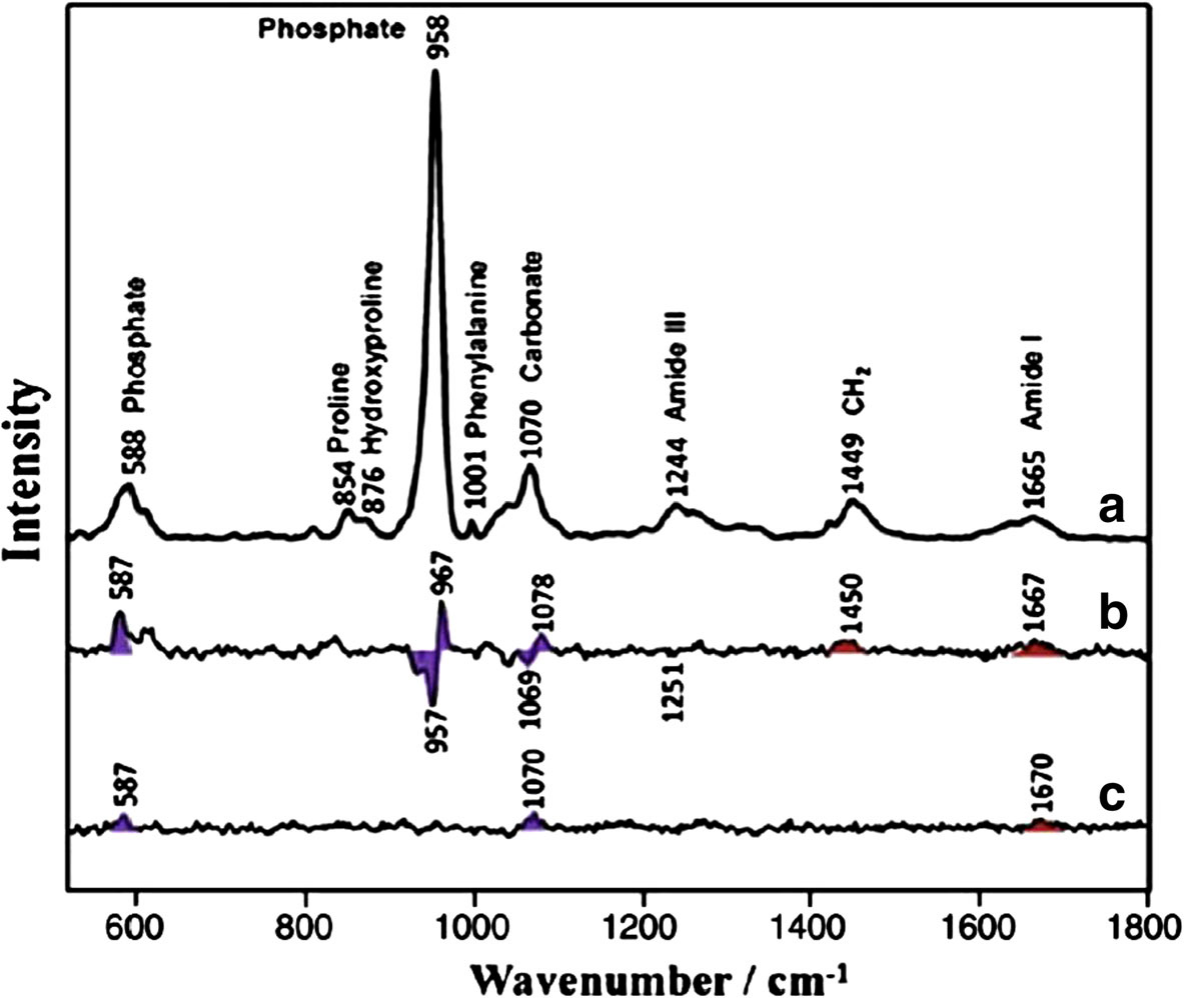
Raman spectra of control (**a**) and Raman difference spectra of [XRT] minus [control] (**b**) and [AMF] minus [control] (**c**). Major bands of the mineral are marked with violet shading. Major bands of the matrix are marked with red shading. (Used with permission. Originally published by Tchanque-Fossuo, et al. 2013 in Raman spectroscopy demonstrates Amifostine induced preservation of bone mineralization patterns in the irradiated murine mandible)

Raman spectroscopy is potentially a very powerful and high-impact technology for application to radiotherapy. It is likely to have a ubiquitous role in the future for measuring treatment effects/response [37, 42, 43]. Figure 2 includes a control Raman spectrum (a) and two Raman difference spectra (b and c), where the control spectrum has been subtracted from two clinical specimens allowing for tissue identification. Indeed there are already early reports for measuring response in oral cancer and lung, breast, and prostate cancer [44, 45]. Raman spectroscopy is also likely to have a critical role in the detection of residual disease post-surgery for guiding post-operative radiotherapy [31]

.

### Elastic scattering spectroscopy (diffuse reflectance spectroscopy)

Elastic scattering spectroscopy (ESS), also known as diffuse reflectance spectroscopy, is a spectroscopic imaging technique that identifies samples of tissue based on their interactions with light [13]. Specifically, ESS measures the absorption and scattering of light from a sample across the visible light spectrum (300–900 nm), which can be used to characterize the composition of a sample [46]. Protein composition, cellular density, hemoglobin content, and hemoglobin percent saturation alter tissue’s absorption and scattering of light. Since many of these tissue characteristics are changed in malignant tissue, they can be used to differentiate benign from malignant tissue [46, 47].

Devices using elastic scattering spectroscopy are being evaluated in clinical settings and showing promising results. In breast tissue samples, ESS was able to discriminate malignant from benign tissues with high sensitivity (81–84%) and specificity (75–89%) [48]. In evaluation of colonic polyps, ESS devices were able to differentiate normal samples, hyperplastic polyps, adenomatous polyps, chronic colitis samples, and colorectal cancer with high sensitivities (77–85%) and specificities 75–88%) [48].

Advantages of ESS-based devices include their ability to be incorporated into handheld devices; rapid data acquisition; lack of need for external fluorophores; and an optical signal that is many times stronger than fluorescence and Raman spectroscopy [13]. Disadvantages of this technology include its propensity to be overwhelmed by operative lighting; blood/hemoglobin signal interference; and variable depth-of-field [13].

**Fig. 3:**
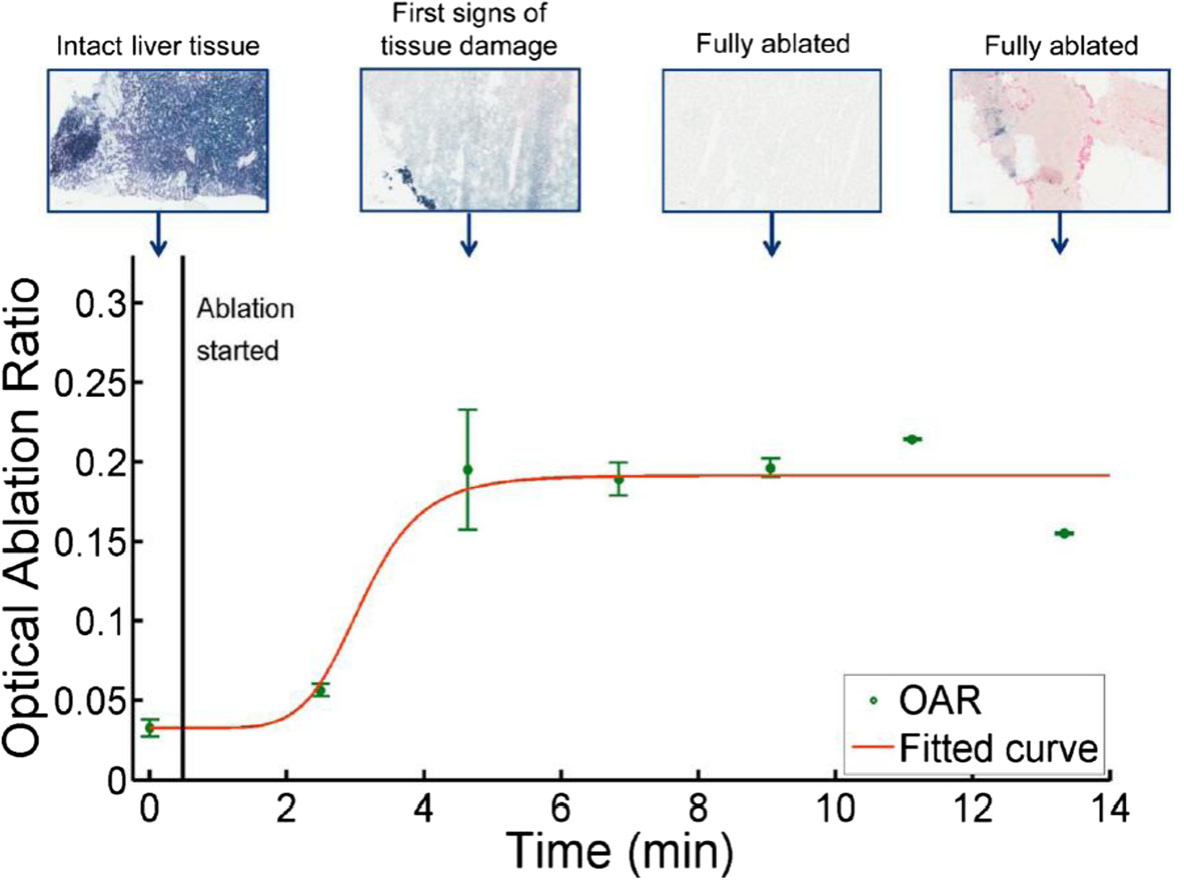
RF ablation monitoring in vivo. Representative graph illustrating the correlation of thermal tissue damage (NADH staining) with dynamic changes in OAR (long distance) measured in vivo during open RF ablation. (Used with permission. Originally published by Tanis, et al. 2017 Real-time in vivo assessment of radiofrequency ablation of human colorectal livermetastases using diffuse reflectance spectroscopy)

Elastic scattering spectroscopy is likely to have significant applications in analysis of surgical margins [such as for breast cancer], detection of radiation/tissue damage, particularly in brain tissue, and monitoring radiotherapy response such as during the radiotherapy course when treating head and neck malignancies [20, 49, 50]. For example, Fig. 3 shows in vivo monitoring of thermal tissue damage following radiofrequency ablation.

### High-frequency ultrasound

Traditional ultrasound (US) imaging has been used in the medical field for many years as a safe and inexpensive imaging modality. Now, the clinical utility of high-frequency US (> 20 MHz) is being explored [51]. US-based devices generate images from the interactions between sound waves and tissue. Because sound wave propagation through a tissue is dependent upon characteristics, such as cell structure, cell density, tissue microstructure, and tissue heterogeneity, ultrasound can be used to differentiate benign from malignant tissues [52].

Currently, high-frequency ultrasound is being evaluated for its utility in breast cancer tumor margin assessment and has shown promising results. In a study that utilized high-frequency US to evaluate surgical margins during invasive breast cancer excision, it was concluded that coupling intraoperative US excision with macroscopic margin assessment via US was safe, useful, efficient, and reduced the number of required reoperations [53].

Strengths of high-frequency US technology include its minimally invasive nature, sub-millimeter resolution, lack of need of fluorescent agents, and existence of approved devices. Disadvantages of this technology include its variance of sensitivity and specificity with different tissues and the need to generate complex, multivariate algorithms for objective image analysis as well as user dependency [13].

High-frequency ultrasound is likely to have a pivotal role in a number of disease sites. Features of ultrasound that make it inherently safe for imaging (due to absence of ionic radiation for diagnosis) and typically portable/hand-held, lends itself to application across many anatomic sites [54]. Potential radiotherapy applications include for superficial tumors and assessment of skin and subcutaneous changes from radiation [55–57]. Ultrasound may also have applications for deeper structures, including uveal melanomas in the setting of proton therapy and prostate radiotherapy [58, 59]. In the case of prostate cancer, both tissue elastography and Doppler ultrasound may have applicability in identifying the neurovascular bundle for avoidance and for monitoring/predicting/reducing erectile dysfunction from prostate radiotherapy.

### Contrast-enhanced ultrasound

Contrast-enhanced ultrasonography (CEUS) uses traditional medical sonography with the addition of ultrasound contrast agents, such as Levovist, Optison, SonoVue, Luminity, for dynamic lesion enhancement patterns that can be appreciated during intermittent and continuous imaging. These blood pool tracers contain microbubbles of nitrogen gas or perfluorocarbon, which have low solubility in blood that allow them have a high degree of echogenicity. This use of microbubble contrast agents with US imaging allows for enhancement of the ultrasound backscatter, which permits production of a sonogram with increased vascular contrast analogous to IV contrast agents used in MRI or CT. These microbubbles also have the ability to be targeted with ligands that can bind specific receptors, allowing for non-invasive means of detecting sites of malignancy [60, 61]. The majority of CEUS applications in current literature discuss its success in detecting liver lesions, but its application has progressively broadened throughout recent years given its value of providing real-time rapid blood flow phenomena.

Some of the main benefits of CEUS are that it is widely-available, safe, has a comparable allergic event incidence to MR contrast compounds, and it has a low incidence of related side effects, including lack of nephrotoxicity or thyroid toxicity. It is also inexpensive and fast, compared to CT and MRI imaging. In addition, several studies including a meta-analysis have shown its ability to characterize lesions with a diagnostic accuracy comparable to MRI and CT [60–62]. Not only does CEUS allow for evaluation of real-time blood flow, but this imaging technique can also provide absolute quantification of tissue perfusion without giving any radiation to the patient. Since the microbubbles can produce robust signals, a lower IV dosage is required compared to MRI contrast agents, for example [60, 61].

The drawbacks of CEUS are similar to other ultrasound-based techniques. As advantageous as the microbubbles may appear, they do not have a long half-life in circulation and are at risk of causing microvascular rupture and hemolysis if the microbubbles degrade in vivo. Other disadvantages can be specific to the site under evaluation. For example, small liver metastases (< 5-10 mm) can be missed with CEUS, as they may be too miniscule to generate visible defects in the portal and late phases. Deeper seated lesions can also be missed due to CEUS’s limited penetration. However, it is worth mentioning that CEUS has shown greater sensitivity than CT and MRI at locating particularly small metastatic lesions [60, 61]. An additional drawback as with other imaging technologies may be user variability.

In general, CEUS has shown to be useful for characterization of lesions in the liver, breast, spleen, prostate, pancreas, and head and neck [62–67]. In addition to detection, this imaging modality can also be utilized for guiding ablations. There continues to be ongoing research on the practical uses of CEUS, with progressive inquiry showing the versatility of this imaging modality and how it can either replace or complement other imaging techniques in oncologic diagnosis and treatment.

### Optical coherence tomography

**Fig. 4:**
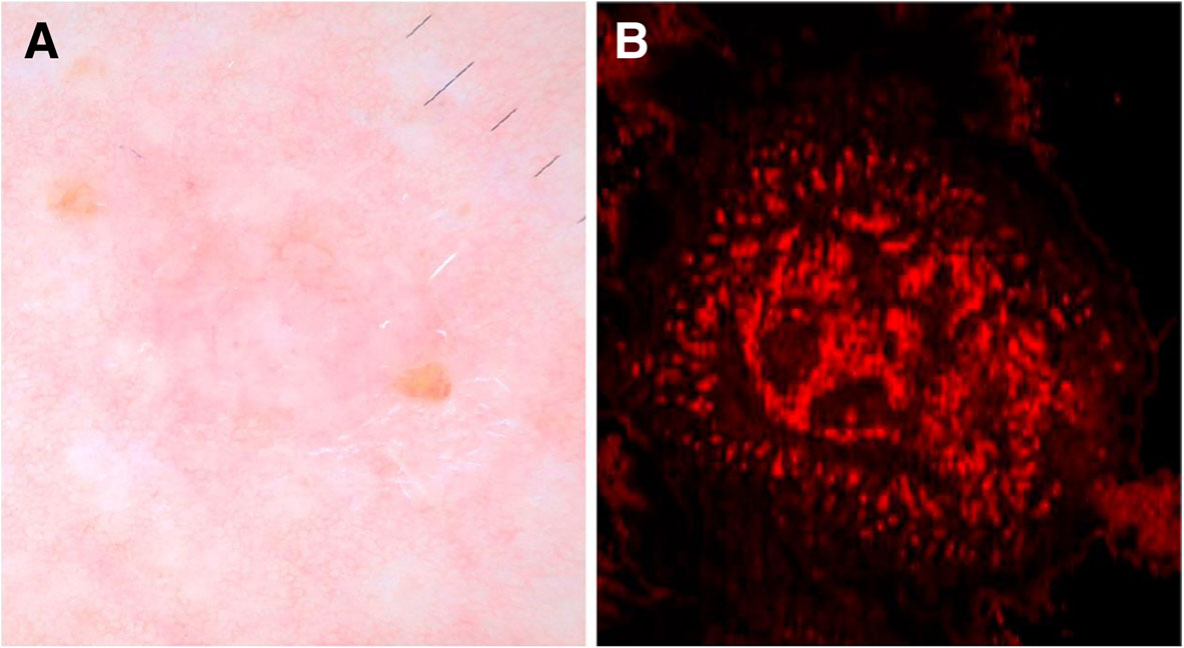
Dermoscopy and en face view of BCC on D-OCT: (**a**) Dermoscopy of BCC with pink-white shiny background, focal ulceration, arborizing vessels. **b** En face D-OCT of BCC shows disarray of thin, irregular vessels that are variable in size compared with the normal facial vessels. In comparison with more aggressive tumors, such as melanoma, the vascular pattern appears confined to the tumor. (Used with permission. Originally published by Levine, et al. 2017 in Optical Coherence Tomography in the Diagnosis of Skin Cancer)

Optical Coherence Tomography (OCT) is an imaging modality that operates upon similar principles as ultrasound but uses near infrared light, instead of sound, to generate images [13, 68, 69]. Specifically, the time delay and intensity of light reflected from a tissue of interest are compared to light reflected from a reference path, and the information is compiled to form a cross-sectional image [13, 68]. Because OCT uses light, rather than sound, it has a much higher resolution than ultrasound [13, 68]. OCT has the potential to have cellular-level resolution, which would allow for visualization of morphologic changes in tissue architecture characteristic of malignancy [13]. For example, Fig. 4 shows a dermoscopic image of basal cell carcinoma beside an image using *en face* optical coherence tomography showing thin, irregular vessels compared to normal facial vessels.

OCT-based applications are being explored over a vast range of applications. OCT devices are already commercially available for evaluation of the eye [70]. In several small studies, OCT has shown promising results in urological applications, such as renal mass identification and prostate cancer surgical margin assessment [69].

Benefits of OCT include its high resolution (10-15um); ability to provide real-time information; be incorporated into handheld devices; lack of need of external fluorophores; and potential for automated image interpretation. A The main limitation of this technology is its shallow depth of field (2 mm) and small field-of-view. [13, 69].

### Optoacoustic imaging

Optoacoustic imaging is a unique imaging modality that generates images using both sound and light. To generate an image, focused light is pulsed over a tissue of interest, and the resulting sound is recorded, which is then compiled into an image [71]. Initial techniques used light of a single wavelength and were used for anatomic imaging, but more recent developments allowing implementation of multiple wavelength light have extended the potential applications of optoacoustic imaging [72].

**Fig. 5:**
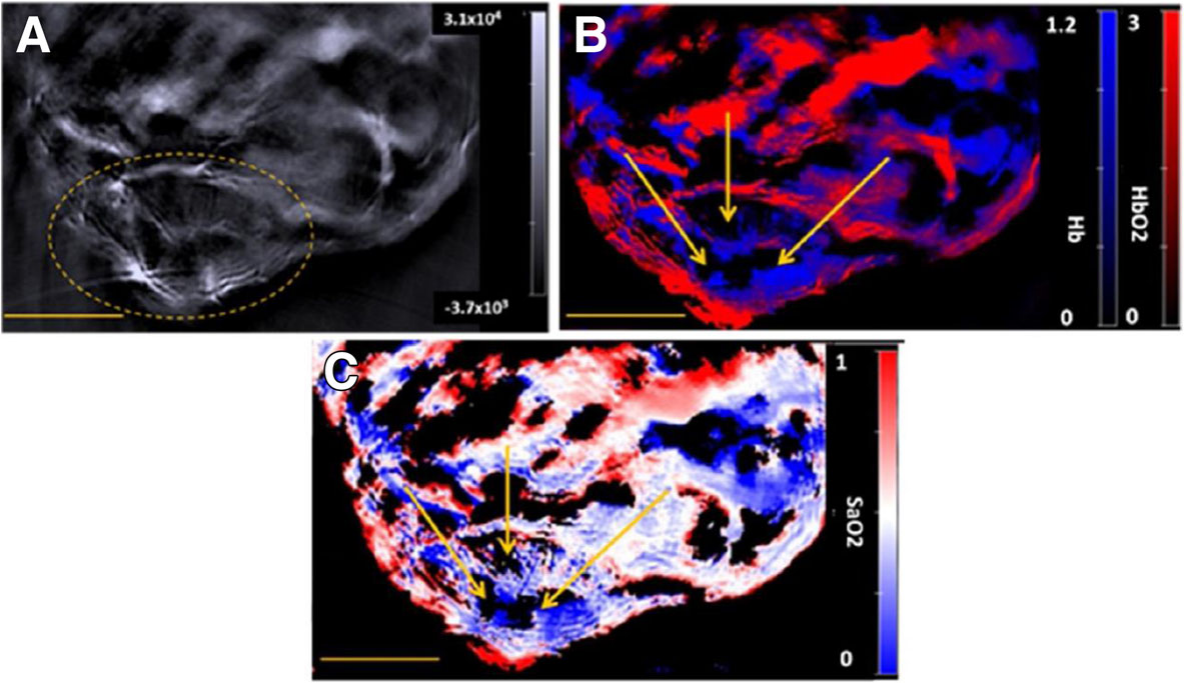
**a** A reconstructed single wavelength MSOT image of mouse M1 (example of a PDA-KPC tumour) at 800 nm. The dashed yellow line indicates the tumour. **b** The corresponding spectrally unmixed image, showing the distribution of Hb and HbO2 components in blue and red respectively. **c** The oxymap image, showing the distribution of the SaO2 values. The yellow scale bar at the lower-left in each of these images is 5 mm. The yellow arrows in images **b** and **c** indicates the centre of a region that lacks any spectral signature of blood. The background, Hb, HbO2 and oxymap colour bars reflect the magnitude of the photoacoustic signals, deoxy-haemoglobin, oxy-haemoglobin and SaO2, respectively, as calculated by the MSOT system. (For interpretation of the references to colour in this figure legend, the reader is referred to the web version of this article.) (Used with permission. Originally published by Shah, et al. 2017 in Value of combining dynamic contrast enhanced ultrasound and optoacoustic tomography for hypoxia imaging)

The potential applications of optoacoustic imaging span many fields. In tissue suspicious of breast cancer, optoacoustic imaging was able to differentiate between cysts and malignancies [72, 73]. For dermatologic applications, a probe has been developed to produce 3D images of the dermis and subcutaneous tissues in real-time. [72, 74]. In one study, optoacoustic technology was combined with ultrasound in an endoscope and used to evaluate the esophagus and colon of rats and rabbits [72, 75]. Lastly, Fig. 5 pictures how the photoacoustic signals from hemoglobin has been used intraoperatively to evaluate tissue perfusion [13, 72]

.

Optoacoustic imaging is an exciting upcoming imaging technology that has immense potential. The advantages of this technology include its potential for real-time imaging; high resolution and improved depth-of-field; and ability to be enhanced with contrast agents, such as targeted fluorophores [13, 71, 72]. Historically, this technology has been limited by a shallow depth-of-field, but with new techniques and advances in technology, it has been improved significantly [13, 71, 72].

Optoacoustic imaging is quite novel and has potential applications in radiotherapy in the context of staging workup, and in the context of planning adjuvant radiotherapy, particularly for patients with residual cancer after surgery [31]. Optoacoustic imaging may also have a unique role in quality assurance for proton therapy [76].

### Confocal microscopy

Confocal microscopy is a non-invasive imaging technique capable of cellular-level resolution [13, 77]. To obtain confocal images, a near-infrared (830 nm), low-power laser is focused on a region of interest called a voxel. Reflected light passes through a pin-hole aperture, which filters out light from undesired locations but allows light from the voxel to pass. Light from the voxel then strikes a photodetector, which is then plotted as a single pixel. Multiple pixels are then stitched together to produce an image [77, 78]. To produce images with larger fields-of-view, images can be stitched together to produce a mosaic [77].

Currently, in vivo clinical applications of confocal microscopy have primarily been in the fields of ophthalmology and dermatology, while other applications are being explored. In ophthalmology, confocal microscopy has been used to “diagnose Acanthmoeba and deep fungal keratitis, [measure]. residual bed thickness… , and [measure]. endothelial cell density” [79]. In dermatology, it has been used in cosmetic studies, dermato-oncology applications, and dermatosis evaluations [77]. Oral applications, such as dysplasia/neoplasia evaluation and management of pathology, have been investigated but further work is needed before it can be incorporated into clinical practice [77].

Benefits of this technology include its micron-level resolution; ability for real-time image acquisition; lack of need of external fluorophores; ability to be enhanced using nonspecific fluorophores; future computer-assisted image analysis; and ability to digitally stain mosaics to resemble hematoxylin and eosin stains [13, 78]. Limitations of this technology include its shallow depth-of-field (200-300um); small field-of-view; high cost of current devices (>$60,000); and additional training needed for manual interpretation [13, 78].

Confocal microscopy is quite early in its development, but radiotherapy applications are potentially for detection and planning of cutaneous tumors (Ibrahim 2014) and soft-tissue sarcomas (Cuneo 2013, Davies 2004) [80–82].

## Biological target volume

The imaging modalities described in this review provide useful information about the biologic behavior of tumors and if incorporated into radiation treatment planning, these modalities may allow for even more personalized radiation plans for individual patients. Further research into the utility of these imaging modalities will shed light on the most ideal set of imaging studies that will aid the Radiation Oncologist in generating target volumes for individual types of tumors. By providing both anatomic and biologic information about the tumor, such imaging modalities may be used to generate a biologic tumor volume (BTV). As described by Ling et al., the BTV incorporates additional information from biological images which can then be used to generate tailored “dose painting” that allows for different doses to be delivered to separate sub-volumes of the tumor (Ling 2000) [83].

Intensity modulated radiation therapy (IMRT), which requires an inverse-planning process, is particularly reliant on the planning target volumes including gross tumor volume (GTV), clinical target volume (CTV), internal target volume (ITV), and planning target volume (PTV), as defined by the International Commission on Radiation Units and Measurements Report No. 50 (ICRU 50) [2]. Likewise, proton therapy is also heavily dependent on imaging and planning target volumes. Thus, a thorough knowledge of the disease process is essential for generating these planning target volumes (ICRU 83) [84]. As such, the additional information used to generate at BTV may allow for more precise and effective delivery of radiation.

Furthermore, because IMRT planning is more conformal than conventional plans, the technique of dose painting can be utilized to deliver different doses to adjacent regions of gross disease and regions at risk for subclinical disease as previously described. For example, when treating tumors in the head and neck region, the gross tumor may be prescribed 70 Gy, a high-risk region may receive 63 Gy, and another low risk region may receive 56–59.4 Gy. This method of delivery is termed simultaneous integrated boost (SIB) and tends to be more conformal than if the doses were delivered sequentially.

The concept of dose painting can be advanced one step further by the inclusion of a BTV. For example, areas of low pO_2_ and high proliferation may be defined and receive higher doses of radiation as compared to the rest of the tumor. Experimental studies have shown that an increase in dose to areas of high ^18^F-FDG uptake had a greater effect on local control than an increased dose to areas with low ^18^F-FDG uptake [85]. Such a strategy may go beyond traditional dose escalation as certain areas of the tumor with aggressive features can be targeted with higher dose radiation [86]. This approach can lead the way for further individualization of cancer treatment with radiation therapy.

Additionally, an exciting utilization of biologic imaging would be the incorporation of such information into the dynamic process of adaptive planning. While current methods rely on imaging to demonstrate anatomic changes during treatment, additional biologic imaging would also provide the Radiation Oncologist with data about the changing microenvironment of the tumor and the resulting changes in BTV during treatment. For example, a Phase I study examining dose escalation in ^18^F-FDG regions and dose painting to voxels based on signal intensity was used for adaptive planning for patients with head and neck cancer [87]. Biologic and anatomical changes based on ^18^F-FDG- PET/CT were used to decrease target volumes. Further research incorporating the concepts of adaptive planning and biologic imaging would work towards increasing the therapeutic ratio of radiation by maximizing dose to the tumor while also limiting dose to the normal tissue thereby curtailing acute and late toxicity.

## Conclusions

In this overview, we have described several up-and-coming imaging technologies that appear to be on the cusp of broader clinical utilization and are being developed at a rapid pace for application to unmet needs in clinical oncology, including radiotherapy. Additionally, for the same reasons these new innovations in imaging technologies may provide beneficial for radiation therapy, the field of oncologic surgery may also find applications for these technologies to enhance diagnostic and therapeutic surgical interventions for cancer patients. Although some, such as Raman spectroscopy, may already have found limited early clinical use in head and neck cancer post-treatment surveillance, it will take concerted investment and clinical trials across many fronts before the most useful modalities are fully validated and widely clinically accepted. In addition, the economic viability of these advances in the developing world will also have to be considered during the testing and trial phases.

Notably, although several of the above modalities listed in the article have early translational data in humans, several, such as those described in the *Near-infrared fluorescent imaging* and *Nonspecific Fluorescent Constrast Agents* sections (and the CW800 and ZW800–1 dyes in particular) only have pre-clinical data at this point. While there are always challenges in reproducing and calibrating approaches used in pre-clinical subjects to human subjects, standard multi-scaler approaches can likely be applied to virtually all of the new technologies listed above to develop them for clinical use [88].

We have presented here an overview of imaging techniques which are likely to have significant future roles over the entire patient care continuum – screening, diagnosis, staging, treatment planning, treatment delivery, and post treatment surveillance. For future reference, we have outlined our findings in Table 1.

**Table 1:** Summary of imaging modalities advantages, disadvantages, and potential applications

MODALITY	Advantages	Disadvantages	Applications
Autofluorescence	Real-time imaging; no fluorophore needed; wide FOV	Shallow DOF; low specificity; high cost	Superficial tumor monitoring; pre-neoplastic evaluation; surgical margin assessment
Near-infrared fluorescent imaging	Real-time imaging; deep DOF; high signal-to-noise	Need of imaging device; dye administration time	Surgical guidance; real-time monitoring and modulation of EBRT dose
Nonspecific Fluorescent Contrast Agents	Low dye toxicity; rapid hepatic clearance of dye	Contraindicated with contrast allergy; low signal-to-noise; no intracellular dye accumulation	Staging; guidance of radiotherapy planning; imaging for treatment delivery
Targeted Contrast Agents	Use Ig-based therapies as ligand; deep DOF via NIRF imaging	Require external fluorophore; few agents currently available	Diagnosis; staging
Radiofrequency Spectroscopy	No fluorophore needed; real-time imaging; handheld devices now exist	Limited FOV; shallow DOF	Residual disease detection; improved diagnostic imaging in solid tumors
Raman Spectroscopy	Real-time imaging; determine tissue response to radiation	Shallow DOF; low signal-to-noise; thermally damage samples	Measuring treatment response; post-op residual disease detection
Elastic Scattering Spectroscopy	Handheld devices; no fluorophore needed; rapid image acquisition; strong optical signal	Signal interference; variable DOF	Radiation damage detection; radiotherapy response monitoring
High-frequency Ultrasound	Sub-millimeter resolution; no fluorophore needed; approved devices	No ionizing radiation; variable sensitivity and specificity; complex image analysis algorithms	Superficial tumor assessment; reduce morbidity of prostate radiotherapy
Contrast-enhanced ultrasound	Real-time, continuous imaging; compatible with targeted contrast agents; widely-available; inexpensive; relatively safe	Shallow DOF; short half-life of contrast; potential for microvascular collapse	Primary/ metastasis characterization; ablation guidance
Optical Coherence Tomography	Cellular-level resolution; real-time imaging; handheld; no fluorophore needed; automated image analysis	Shallow DOF; narrow FOV	Renal mass identification; prostate cancer margin assessment
Optoacoustic Imaging	Real-time imaging; high resolution; enhanced by contrast agents	Historically shallow DOF but now improved	Staging; post-op planning for residual disease
Confocal Microscopy	Micron-level resolution; real-time imaging; no fluorophore needed; enhanced with fluorophores; automated image analysis; digital staining	Shallow DOF; narrow FOV; high device cost; user training necessary for manual interpretation	Detection and planning of cutaneous tumors and soft-tissue sarcomas

## Abbreviations

BTV: Biologic tumor volume
CEUS: Contrast-enhanced ultrasound
CT: Computed tomography
CTV: Clinical treatment volume
EPR: Enhanced permeation and retention
ESS: Elastic scattering spectroscopy
GTV: Gross tumor volume
ICG: Indocyanine green
ICRU: International Commission on Radiation Units and Measurements
IMRT: Intensity modulated radiotherapy
ITV: Internal target volume
LMIC: Lower-middle income countries
MB: Methylene blue
MRI: Magnetic resonance imaging
NIRF: Near-infrared fluorophore
OAR: Organs at risk
OCT: Optical coherence tomography
PET: Positron emission tomography
PTV: Planning target volume
RS: Radiofrequency spectroscopy
US: Ultrasound
